# Fractionation gives therapeutic benefit in animal model of [^177^Lu]Lu-PSMA-617 therapy of prostate cancer

**DOI:** 10.1186/s13550-026-01417-9

**Published:** 2026-03-25

**Authors:** Oskar Vilhelmsson Timmermand, Axel Östholm, Wahed Zedan, Joanna Strand, Mohamed Altai, Anders Örbom

**Affiliations:** Division of Oncology, Department of Clinical Sciences, Lund University, Lund, Sweden

**Keywords:** Prostate cancer, PSMA-617, Radionuclide therapy, Hyperfractionation, Fractionation, Animal model

## Abstract

**Background:**

The clinical standard practice of [^177^Lu]Lu-PSMA-617 therapy is a single injection per treatment cycle, with 6 weeks between cycles. While clinical schedules currently utilize single-bolus cycles, splitting a treatment cycle into multiple smaller injections has demonstrated benefits in other radionuclide therapies, preclinically and in clinical studies. Potential mechanisms for improved therapeutic efficacy include receptor recycling, receptor upregulation, or targeting new cell growth between fractions. This study aims to investigate the effects on tumor size and animal survival, in a mouse model of prostate cancer, of fractionating [^177^Lu]Lu-PSMA-617 therapy compared to the same total activity in a single injection.

**Results:**

BALB/c mice bearing subcutaneous LNCaP prostate cancer tumors, below 650 mm^3^ in volume, were treated with either 1 × 30 MBq, 2 × 15 MBq (24-hour window), or 2 × 15 MBq (6-day window). SPECT/CT imaging showed a higher, but not significantly so, tumor uptake in the 24-hour window group than in the unfractionated one. Differences in tumor sizes were primarily visible during regrowth after therapy, with significantly smaller relative tumor sizes in the 24-hour window group compared to the unfractionated group day 89–95 post inoculation. The median survival for the 24-hour group (71.5 days) was significantly longer than that of the unfractionated group (46 days; *p* = 0.024). The 6-day group tumor sizes and survival came close to the 24-hour one, but was not significantly better than the unfractionated group.

**Conclusion:**

This study demonstrates that fractionation gives therapeutic benefit in an animal model of [^177^Lu]Lu-PSMA-617 therapy of prostate cancer for tumors in this size range. A shorter 24-hour window outperformed a longer of 6 d between fractions. The outlook for clinical translation will depend on if the mechanism is relevant at conditions, blood ligand concentration etc., that differs between the animal model and human patients.

**Supplementary Information:**

The online version contains supplementary material available at 10.1186/s13550-026-01417-9.

## Background

[^177^Lu]Lu-PSMA-617 (Pluvicto™) is the first FDA- and EMA-approved radioligand therapy for Prostate specific membrane antigen (PSMA)-positive metastatic prostate cancer (PCa). The approved schedule involves a single injection every 6 weeks for up to 6 doses [1, 2]. Although radionuclide therapy can be considered hyperfractionated in external beam radiation therapy parlance with the moments between radionuclide decays serving as fractionation windows, the term “fractionated” typically implies splitting a single treatment cycle into multiple injections [3].

In radionuclide therapy for neuroendocrine tumors, fractionating [^177^Lu]Lu-Octreotate has been shown to delay tumor regrowth and prolong survival in animal models [4, 5]. The proposed mechanisms for this are the recycling of cell-surface receptors between fractions, allowing more to become available for subsequent doses, and the upregulation of receptor expression caused by radiation from the first fraction. Both theories assume that a single, large dose saturates the tumor’s receptors. Saturation has also been observed for PSMA in patients up to at least 2 h p.i [6].

In prostate cancer, splitting a treatment cycle of anti-PSMA antibody [^177^Lu]Lu-J591 into two fractions over two weeks allowed for higher total activity without prohibitive hematologic toxicity [7, 8]. For [^177^Lu]Lu-PSMA-617, clinical dose escalation studies using a two-week fractionation window have been reported [9]. Zheng et al. (2024) found that in a mouse model, 3 × 7.4 MBq of [^177^Lu]Lu-PSMA-617 yielded higher tumor uptake and cumulative absorbed dose over 2.5 days compared to 1 × 37 MBq [10]. Benefits of fractionation over single injection has recently been shown pre-clinically with [^212^Pb]Pb-AB001, another small-molecule PSMA-targeting ligand labelled with an alpha emitter [11].

PSMA is assumed to recycle within minutes of ligand internalization, similar to the transferrin receptor [12]. This allows for continued binding of [^177^Lu]Lu-PSMA-617, which circulates in the blood of mouse models for at least 24 h post-injection [13, 14]. Irradiation-induced PSMA upregulation in PCa cells has been demonstrated [15], although this effect may be transient [16]. While the ligand’s blood concentration is a vital parameter for these mechanisms, the clinical specific activity of [^177^Lu]Lu-PSMA-617 is uncertain, with estimates ranging from 48 to 88 GBq/µmol [17]. We know from studies of mouse xenografts using RM1-hPSMA tumor cells, that with a specific activity of 62 MBq/nmol, an injection of 15 MBq gave around 40% higher uptake than 31 MBq [18].

**Fig. 1:**
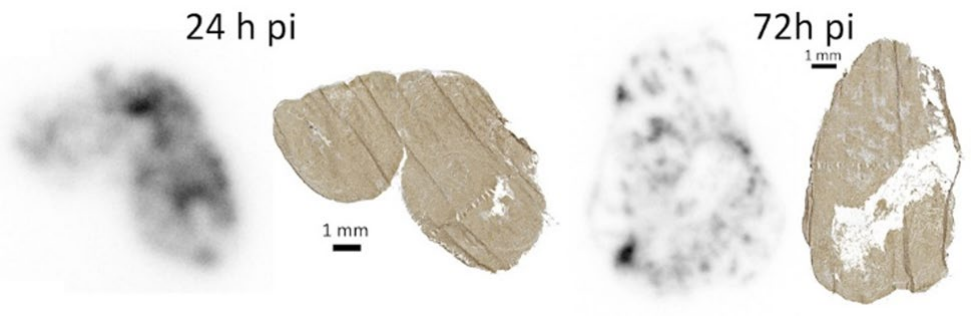
Autoradiography images, individually scaled from zero (white) to maximum (black) activity, of tumor sections of PC-3 pip xenografts from mice injected with [^177^Lu]Lu-PSMA-617 since two different durations, and adjacent sections from the same tumors, immunohistochemically stained for expression of PSMA. Note areas already at 24 h p.i., and increasingly at 72 h p.i., with expression of PSMA but very low accumulated radioactivity. Methodology detailed in [20]

One phenomenon that could affect the efficacy of fractionation is the very complex effect of radiation on tumor vasculature, with low doses promoting angiogenesis and high doses inhibiting it, even though high doses also increase vascular permeability [19]. Fractionating doses could potentially impair, but also improve, delivery of subsequent doses. Another, with implications for treatment efficacy, is that subsequent treatment fractions could target newly grown tumor cells. This theory is supported by our research showing that already 24 h post-injection of [^177^Lu]Lu-PSMA-617, and increasingly afterwards, tumors contain viable, PSMA-expressing cells without radioactivity uptake [20], as illustrated in Fig. 1.

This study aims to investigate the long-term therapeutic effects of fractionating, using different time intervals, [^177^Lu]Lu-PSMA-617 therapy on tumor size and survival in a PCa animal model.

## Results

### Radiolabeling

Labeling of PSMA-617 resulted in a radiochemical yield of 99.9 ± 0.1%, and therefore no further purification was required prior to injection into animals. ITLC measurement in Supplementary Fig. 1.

### Activity uptake, SPECT/CT

**Fig. 2:**
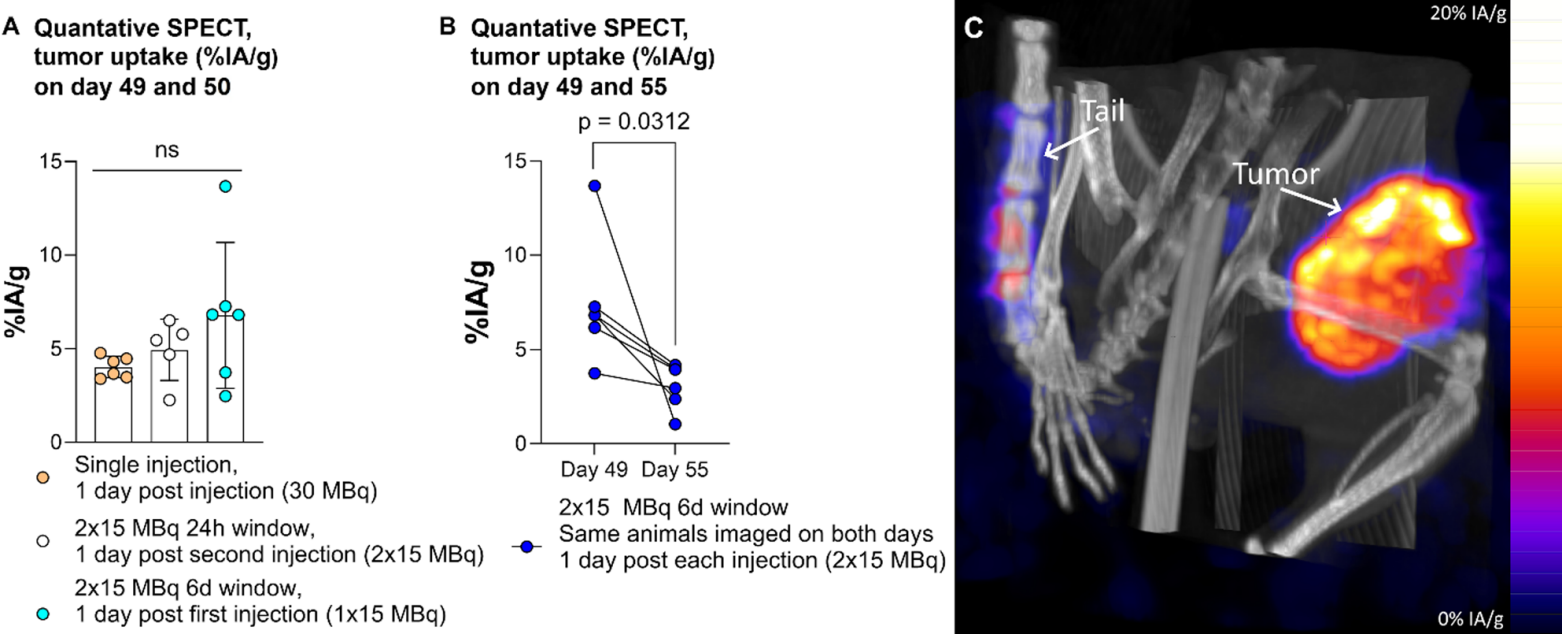
Quantitative SPECT, tumor uptake of [^177^Lu]Lu-PSMA-617 (%IA/g) at (**A**) 49 and 50 days post inoculation (1 day p.i. for single injection, for the last injection of the 2 × 15 MBq 24-hour window group, and the first injection of the 2 × 15 MBq 6-day window group), and (**B**) 49 and 50 days post inoculation (pairwise comparison of uptake in animals scanned 1 d p.i. both the first and second injection in the 2 × 15 MBq 6-day window group, note that the second time point is calculated as percentage of all activity injected so far, corrected for physical decay. (**C**) Representative maximum intensity projection SPECT/CT image of a 24-hour fractionation group animal 24 h post second injection. Note activity uptake in the tumor and some residual activity at the injection site in the tail

Initial tumor activity uptake (%IA/g of all activity injected up until imaging), presented in Fig. 2A, showed no statistically significant differences between groups. However, the group with 6-days between fractionations had significantly lower [^177^Lu]Lu-PSMA-617 accumulation 1 day post the last 15 MBq injection compared to 1 day post the first (Fig. 2B). One day post the last injection, the 1-day group’s median uptake was within 80% of the 6-day group’s initial median uptake, despite having received 2 × 15 MBq. The unfractionated group (1 × 30 MBq) showed only 59% of that initial median.

### Tumor sizes

**Fig. 3:**
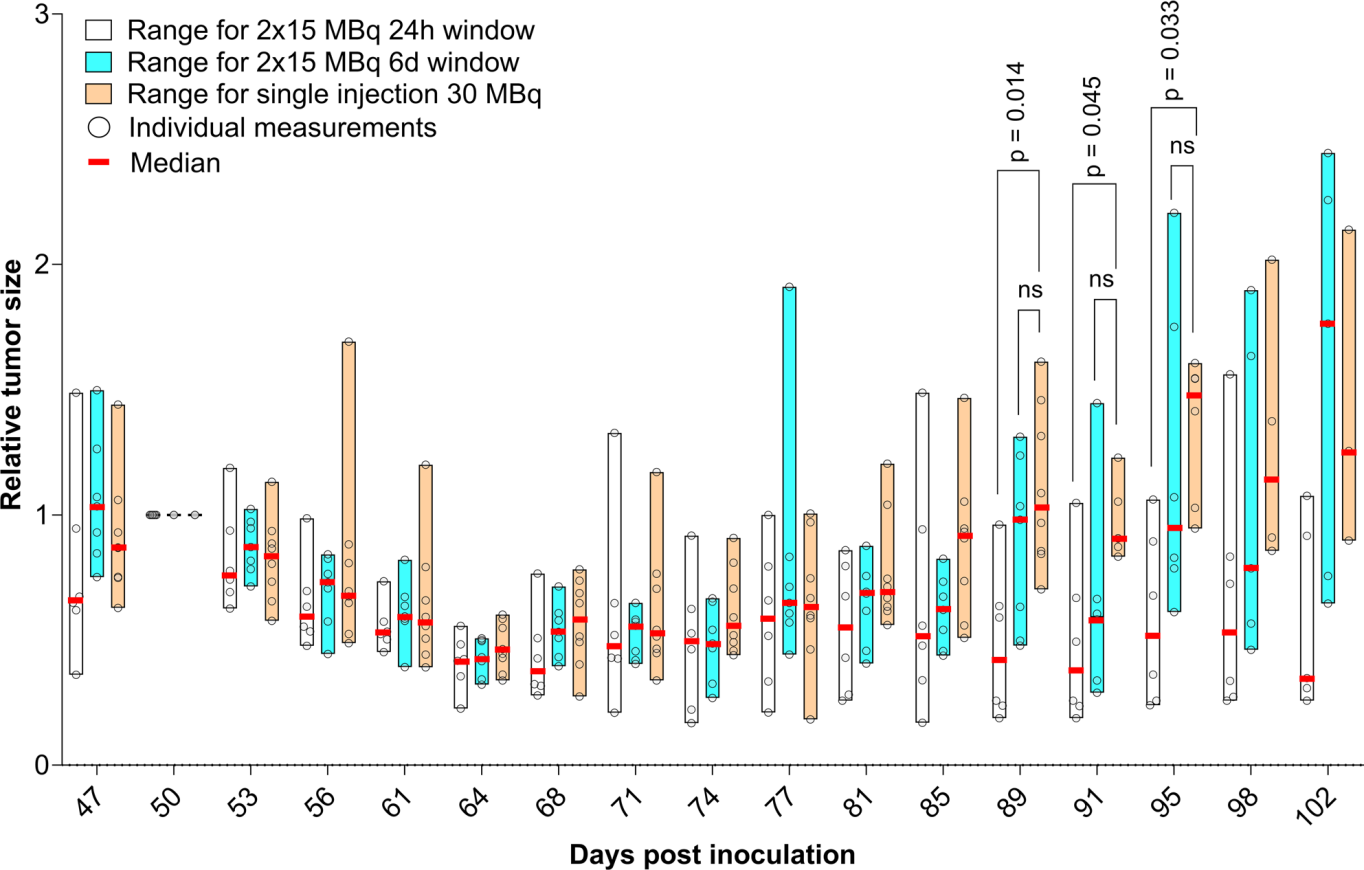
Median, range, and individual values of relative tumor size, normalized per animal to day 50 post inoculation. Injections of 24 h-group on day 48 and 49, of 6 d-group on day 48 and 54, and single injection on day 49. Note that x-axis only extends for as long as each group has at least 3 surviving animals

To adjust for variations in initial tumor size, relative tumor sizes were calculated from day 50 post-inoculation (peak pre-therapy size). These are presented in Fig. 3, with absolute data in Supplementary Fig. 2. From day 85 onwards, the unfractionated group’s median relative tumor size was consistently at least 177% larger than the 1-day group. Significant differences in relative tumor sizes were observed between these groups on day 89 (*p* = 0.014), day 91 (*p* = 0.045), and day 95 (*p* = 0.033). These results hold for comparisons of tumors below 650 mm^3^; for larger tumors (up to 750 mm^3^), the resulting cohorts exhibit significant differences in relative tumor volume only at day 89 while the survival advantage of the 1-day fractionation group only remains when including the animal sacrificed due to poor health (see Supplementary material). This possibly indicates that larger tumors require higher activity per injection for control [21].

### Survival

**Fig. 4:**
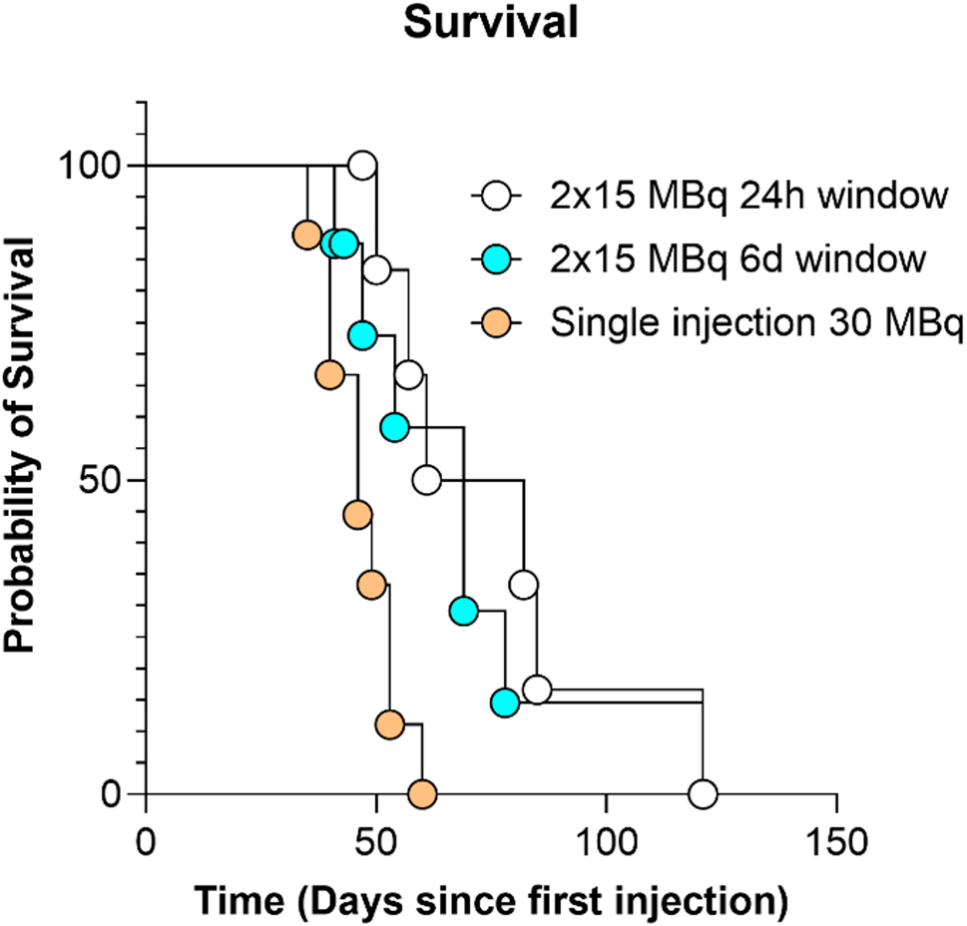
Probability of survival per group. Note that end of study was on day 120

Animals were sacrificed primarily due to tumor size; one unfractionated animal was excluded due to poor health. Survival data (Fig. 4) showed median survival times after the first injection for the 1-day group, 71.5 (range 50–121) days, were significantly (*p* = 0.024) longer than for the unfractionated group, 46 (35–60) days. With 69 (41–121) days for the 6-day group.

## Discussion

Fractionated 2 × 15 MBq dosing (24-hour interval) significantly improved animal survival and delayed tumor growth versus a single 30 MBq injection. Although it is outside the scope of this study to determine the exact mechanism of the positive effect from fractionation, we can speculate. While our uptake levels of [^177^Lu]Lu-PSMA-617 depending on fractionation trend in the same direction, they are not significantly higher with fractionation like Zheng et al. (published after our study was initiated), who used smaller fractions and a different tumor model [10]. For our study, we cannot say with confidence that the difference in survival and tumor growth depends on differences in tumor uptake. Our finding that fractionation is beneficial corresponds to the PSMA-targeted alpha therapy study on [^212^Pb]Pb-AB001. In that case a 7-day window was better than a 3-day one, indicating that the most optimal fractionation schedule could depend on the specific ligand, radionuclide etc. [11].

That fractionation 2 × 15 MBq with a 24-hour window result in similar level of uptake (%IA/g) as 1 × 15 MBq might be because receptors once again become available at or before 24 h. A shorter window might even be feasible given the proposed minute-scale receptor recycling time [12], something which could be investigated by determining the blocking duration in vitro. The previous fraction is unlikely to occupy receptors at 24 h as according to published data, blood content in mice is low at 24 h (0.002%IA/g) and undetectable at 48 h p.i [13]. Our choice of 30 MBq as the total injected activity was partly due to it being a common activity for PSMA-targeted ^177^Lu therapy studies [18, 22, 23], and partly due to a desire that each fraction having at least some therapeutic effect on its own [24]. While Fendler et al. showed a 40% higher xenograft tumor uptake using 15 MBq rather than 31 MBq injections with similar specific activity to us, our study used a cell line with an order of magnitude greater PSMA-expression than their model, so a-priory we would not have expected our 30 MBq activity to saturate the LNCaP tumor [18].

The reduced efficacy of the 6-day fractionation versus the 24-hour one suggests a complex relationship between different factors for successful fractionation and that the effect isn’t solely due to treatment of new tumor growth, as that growth increases over time [20]. However, if this mechanism applies clinically, fractionation in radionuclide therapy, similar to hyperfractionation in external radiotherapy, could be made to benefit patients with rapid tumor growth [25]. Less efficient 6-day fractionation might stem from high absorbed doses to tumor vasculature, impairing the second fraction’s delivery [19]. The observed lower uptake post-second injection (Fig. 2B) could indicate either reduced tumor penetration of, or target availability for, the second fraction, or loss of activity from the first fraction due to macrophage removal of dead cells [26]. We have not studied the effect of fractionation on normal tissue, however if the cause of increased therapeutic effect is due to increased dose stemming from PSMA receptor mechanisms then that might also affect PSMA-expressing structures such as proximal renal tubules but not organs with non-specific uptake [27]. Less injected ligand might also lead to worse tumor to normal organ uptake ratios, which in therapy against neuroendocrine tumors are improved by pre-dosing with cold ligand [28]. Our formula of calculating the tumor size where we use half the tumors measured width as the depth diameter of the ellipsoid is due to our group’s prior research which found assuming depth equals full width overestimates volume by 150% [29]. We did not have access to ultrasound or MRI to measure tumor sizes in this study.

The question remains if this positive effect of fractionation can be translated to the clinic. Caution is advised since time-dependent strategies may differ in effectiveness between mice and man due to difference in systemic clearance and distribution profile [30]. Clinical studies of PSMA-targeting antibodies and [177Lu]Lu-PSMA-617 have prioritized activity escalation, not comparing fractionated and unfractionated equal total-activity schedules [7–9]. One weakness of our animal model is that the activity/blood volume or activity/body mass (30 MBq/2 mL/24 g) is over 10 times higher for blood volume and almost 15 times higher for body mass than human standard treatment (7.4 GBq/5 L/88 kg). If fractionation’s benefit stems from high receptor occupancy versus recycling, blood ligand concentration is critical, as competitive inhibition is clinically observed [6]. Clinical investigation of blocking duration and tumor penetration with diagnostic tracers could avoid excessive patient doses. A clinical translation requires sufficient benefit to justify additional treatment costs [31], and with our results as well as others [11] showing benefit from fractionation at the same total activity, at least the radiopharmaceutical cost could be kept constant.

## Conclusion

This study demonstrates that, in principle, fractionation gives therapeutic benefit in an animal model of [^177^Lu]Lu-PSMA-617 therapy of prostate cancer. However, the timing of the second dose matters with only the shorter window between fractions significantly outperforming the single dose. Further research into the mechanism and size of the effect will determine the feasibility of translation to the clinic.

## Methods

### Animal model

LNCaP cells (ATCC, Manassas, VA, USA) were cultured in RPMI 1640 medium (Thermo Scientific, Waltham, MA, USA) supplemented with 10% foetal bovine serum (Thermo Scientific) and antibiotics (Thermo Scientific). Cells were maintained at 37 °C, 5% CO2, detached with trypsin-EDTA (Thermo Scientific), and regularly tested for Mycoplasma.

Day 0: 7–8 week old BALB/c nude male mice (Janvier Labs) were inoculated subcutaneously with 5 million LNCaP cells in 200 µL 1:1 RPMI-1640/Matrigel (BD Biosciences, Bedford, MA, USA).

Tumor volumes were measured twice weekly using calipers, calculated as an ellipsoid (width, length, and half width). Animals were weighed weekly from day 48 post-inoculation. Sacrifice occurred if tumor length/width exceeded 15 mm, volume exceeded 1000 mm^3^, or general health severely deteriorated.

### Radiolabeling

Lyophilized PSMA-617 (MedChemExpress, Monmouth Junction, NJ, USA) was reconstituted in chelexed 0.2 M ammonium acetate buffer (pH 5.5) to 2 µg/µL. No-carrier-added ^177^LuCl₃ (186–885 MBq) (ITM GmbH, Germany) was added to PSMA-617 (specific activity 51.1–64.6 MBq/nmol) and incubated at 90 °C with vortexing for 30 min. The final solution was diluted with 1% bovine serum albumin (BSA) in phosphate-buffered saline (PBS) to minimize radiolysis.

### Therapy study

On day 47 post-inoculation, a total of 22 animals with tumors below 650 mm^3^ were grouped: 1-day fractionation (*n* = 6, 360 mm^3^ mean), 6-day fractionation (*n* = 7, 389 mm^3^), and unfractionated (*n* = 9, 400 mm^3^). One animal from the 1-day group (initially *n* = 7) was subsequently excluded due to misidentified tumor absence.

Animals received i.v. in the tail vein 15 MBq ^177^Lu-PSMA-617 on days 48 and 49 (1-day fractionation), or days 48 and 54 (6-day fractionation), or 30 MBq on day 49 (unfractionated).

### SPECT imaging

Tumor uptake SPECT/CT imaging was performed for subsets of animals. This occurred on day 49 (*n* = 6) for the 6-day fractionation group, on day 50 (*n* = 5) for the 1-day fractionation group, and the unfractionated group (*n* = 6), and on day 55 (*n* = 5) for the 6-day fractionation group. For the 6-day fractionation group, 5 of the animals that were imaged on day 49 were also imaged on day 55. Animals were anesthetized with 2–3% Isoflurane (Baxter, Deerfield, IL, USA). Imaging utilized a nanoScan^®^ SPECT/CT (Mediso, Budapest, Hungary) system. Mice were imaged for 20 min using a standard single-energy window sequence (188–229 keV). A single mouse bed/APT62 collimator was used on days 49/50, and a triple mouse bed/APT63 collimator on day 55 to fit the allotted instrument time that day. The system was calibrated against a large diameter syringe with a known activity of ^177^Lu directly before each day of imaging. CT images provided attenuation correction and anatomical reference. SPECT images were reconstructed with Tera-Tomo™ 3D (Mediso) using the default standard quality settings. Tumor activity uptake was quantified by manual VOI drawing using VivoQuant 2021 (Invicro, Boston, MA, USA) as a percentage of all injected activity so far (%IA/g), corrected for tail activity and physical decay, assuming 1 g/mm^3^ density.

### Statistics

Given the limited sample size, non-parametric tests were employed. The independent-samples Kruskal-Wallis test with Bonferroni correction was used. Analysis was performed via IBM SPSS Statistics (version 27; IBM, Armonk, NY, USA). P-values below 0.05 were considered significant.

## Supplementary Information

Supplementary Material 1

## Data Availability

The datasets used and/or analysed during the current study are available from the corresponding author on reasonable request.

## Abbreviations

PSMA: Prostate-specific membrane antigen
PCa: Prostate cancer
SPECT: Single photon emission computed tomography
CT: Computed tomography
IA: Injected activity
BSA: Bovine serum albumin
PBS: Phosphate-buffered saline
