# Supplementary material for: Fractionation gives therapeutic benefit in animal model of [177Lu]Lu-PSMA-617 therapy of prostate cancer
Source: EJNMMI Res. 2026 Mar 25;16:47. doi: 10.1186/s13550-026-01417-9 (PMC13022095; doi:10.1186/s13550-026-01417-9)
Supplement: Supplementary file 1 — Supplementary Material 1 [file 13550_2026_1417_MOESM1_ESM.pdf]

Supplementary material – Fractionation gives therapeutic benefit in animal model of [^{177}Lu]Lu-PSMA-617 therapy of prostate cancer

Oskar Vilhelmsson Timmermand, Axel Östholm, Wahed Zedan, Joanna Strand, Mohamed Altai, Anders Örbom

Effect of tumor size at start of therapy

If instead of selecting 650 mm^3 as the limit on tumor size on day 47 post inoculation, we instead include all remaining animals with tumors of a volume below below 750 mm^3 then the three groups become: 1-day fractionation (n=7, 451 mm^3 mean tumor size), 6-day fractionation (n=8, 394 mm^3) and unfractionated (n=9, 400 mm^3). Including these animals makes day 89 the only day with is a statistically significant differences in relative tumor sizes between the 1-day fractionation group and the unfractionated group (p=0.008). The statistically significant longer survival of the 1-day fractionation group compared to the unfractionated group remains if you include the unfractionated animal sacrificed due to poor condition at 35 days post first injection (p=0.032), but not if you do not include it.

Supplementary figures

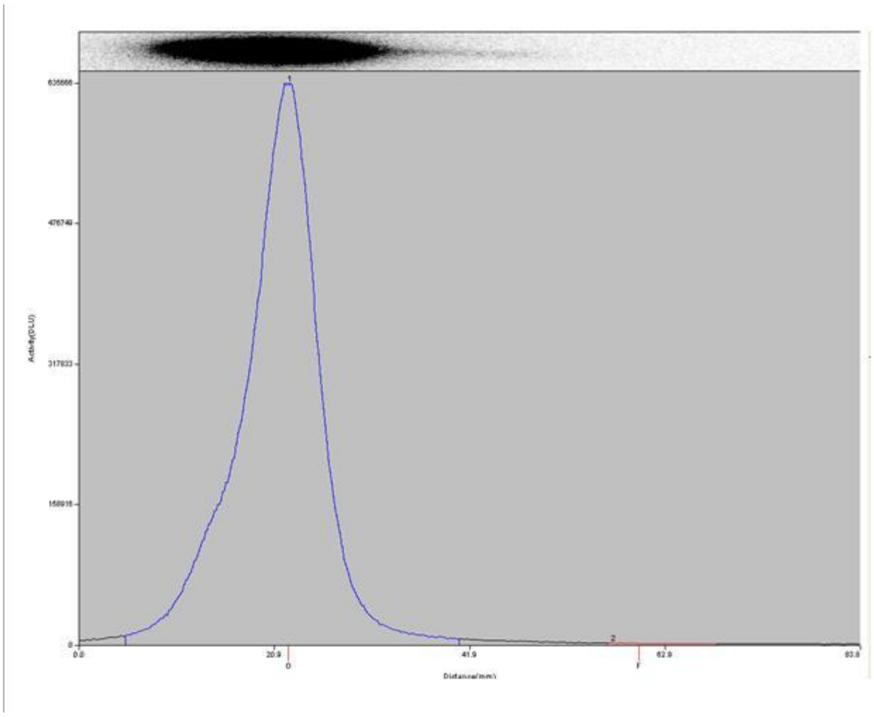

Supplementary figure 1. ITLC measurement from the labelling of PSMA-617 with ^{177}Lu with the large peak representing the labelled [^{177}Lu]Lu-PSMA-617 compound.

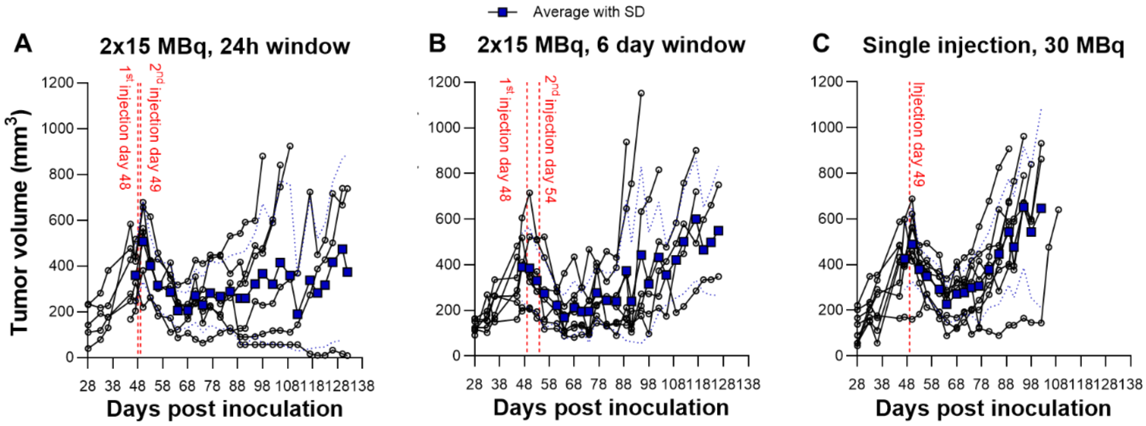

Supplementary figure 2. Tumor volume for each animal in the different groups with injection day(s) as well as average (blue square) and standard deviation (blue dotted line).
